# Design and Integration of Electrical Bio-impedance Sensing in Surgical Robotic Tools for Tissue Identification and Display

**DOI:** 10.3389/frobt.2019.00055

**Published:** 2019-07-17

**Authors:** Zhuoqi Cheng, Diego Dall'Alba, Simone Foti, Andrea Mariani, Thibaud Chupin, Darwin G. Caldwell, Giancarlo Ferrigno, Elena De Momi, Leonardo S. Mattos, Paolo Fiorini

**Affiliations:** ^1^Department of Advanced Robotics, Istituto Italiano di Tecnologia, Genova, Italy; ^2^Altair Robotic Labs, Department of Computer Science, University of Verona, Verona, Italy; ^3^NearLab, Electronic Information and Bioengineering Department, Politecnico di Milano, Milan, Italy

**Keywords:** electrical bio-impedance, tissue identification, da Vinci Research Kit, user interface, intra-operative sensing, augmented reality

## Abstract

The integration of intra-operative sensors into surgical robots is a hot research topic since this can significantly facilitate complex surgical procedures by enhancing surgical awareness with real-time tissue information. However, currently available intra-operative sensing technologies are mainly based on image processing and force feedback, which normally require heavy computation or complicated hardware modifications of existing surgical tools. This paper presents the design and integration of electrical bio-impedance sensing into a commercial surgical robot tool, leading to the creation of a novel smart instrument that allows the identification of tissues by simply touching them. In addition, an advanced user interface is designed to provide guidance during the use of the system and to allow augmented-reality visualization of the tissue identification results. The proposed system imposes minor hardware modifications to an existing surgical tool, but adds the capability to provide a wealth of data about the tissue being manipulated. This has great potential to allow the surgeon (or an autonomous robotic system) to better understand the surgical environment. To evaluate the system, a series of *ex-vivo* experiments were conducted. The experimental results demonstrate that the proposed sensing system can successfully identify different tissue types with 100% classification accuracy. In addition, the user interface was shown to effectively and intuitively guide the user to measure the electrical impedance of the target tissue, presenting the identification results as augmented-reality markers for simple and immediate recognition.

## 1. Introduction

Robot-assisted Minimally Invasive Surgery (RMIS) has come to the forefront in the last decades since this technology can provide enhanced dexterity and 3D perception of the surgical field. These advantages produce a surgical approach that is more ergonomic for surgeons and safer for patients, as described in Elhage et al. (2007). Specifically, RMIS allows the surgeon to access small and complex anatomical districts (e.g., digestive tract), and perform surgery with reduced invasiveness and in a more intuitive and confident way according to Pavan et al. (2016). Additionally and consequently, the patient can benefit from lower occurrence of intra and post operative complications, shorter hospitalization, less pain, and faster recovery.

The incorporation of real-time sensing technologies during complex medical procedures is an essential component for novel surgical robotic platforms. In fact, vision is currently the only sensing technology that provides real-time feedback from the surgical site in most of the existing surgical robotic systems (such as the da Vinci robot by Intuitive Surgical Inc., Sunnyvale, California, USA). However, it is challenging for the surgeon to recognize different tissues in the surgical field due to the fact that the visual properties of most organs are very similar from the endoscopic camera, especially when the field of view is under poor illumination conditions, occluded by smoke produced during electrocautery or by surgical tools. As reported by Penza et al. (2014), additional assistive technologies such as multi-modal data registration and image guided navigation systems are very helpful in these complex conditions. Also, a study by Katić et al. (2016) introduced a method for providing surgical context awareness by identifying surgical activity and retrieving anatomical structures in the image. Alternatively, another study by Moccia et al. (2018) proposed to use deep learning approach for identifying different tissue types in endoscopic video images. However, the identification accuracy can be significantly affected by the illumination condition of the target. In addition, such technologies commonly require heavy computation which also limits their application in realistic surgical conditions.

To address this difficulty, intra-operative sensing technologies have been developed to ensure safer and faster RMIS procedures. For instance, a force sensor was developed and integrated into an articulated surgical tool for proving haptic feedback to the surgeon (Konstantinova et al., 2014). However, in addition to the complexity of the sensor design, this technology requires heavy modifications to existing surgical tools, as well as complicated control schemes to feedback the haptic signal without making the system unstable.

Despite the specific type of advanced sensing technology, the introduction of novel sensing requires proper integration to the standard surgical interface such that the surgeon can easily read and interpret the sensing information or medical instructions. Simorov et al. (2012) described that the user interface needs to be merged with the surgeon console of the robotic system in an ergonomic and comfortable way, without interfering with surgeon's perception or requiring complex training. However, the design of such a user interface can be difficult since the add-in information to the interface should avoid distractions and prevent an increase of the surgeon's cognitive overload, which was demonstrated to be already high during critical steps of a complex surgical procedure by Guru et al. (2015). A good example of such advanced sensing and user interface integration is the incorporation of the Firefly fluorescence imaging into the da Vinci surgical robotic system. The feature based on venous injection of a contrast agent (indocyanine green) has been introduced to provide enhanced visualization for discrimination of vessels and anomalies in soft tissue perfusion. Its user interface allows the surgeon to toggle the view between normal illumination and fluorescence imaging mode via the surgeon console (Meershoek et al., 2018).

In consideration of the limitations of the state-of-the-art work presented above, we propose a novel sensing system for intra-operative tissue identification, which includes an Electrical Bio-Impedance (EBI) sensor and an Advanced User Interface (AUI). The EBI sensing technology is used in this study, which has demonstrated to provide significant improvements to various surgical procedures such as in Kalvøy and Sauter (2016), Cheng et al. (2017), and Schoevaerdts et al. (2018). An EBI sensor is designed and integrated into a standard bipolar tool for providing fast and accurate tissue detection and identification. In addition, the AUI is designed and merged to the surgeon console to provide the surgeon with augmented reality (AR) visualization of additional information about the surgical scene. In addition, the kinematics information retrieved from the robotic system is also displayed on the AUI for guiding the EBI measurements and tissue classification.

The rest of the paper is organized as follows. Section 2 provides details about the EBI sensor and the AUI, as well as a description of their integration into a surgical robot platform. Section 3 presents the experiments to assess the proposed system in terms of tissue identification accuracy and AUI mark position accuracy. Then, a discussion of the experimental results is provided in section 4. Finally, section 5 draws conclusions and presents future directions of this work.

## 2. The System Design

### 2.1. System Overview

**Figure 1:**
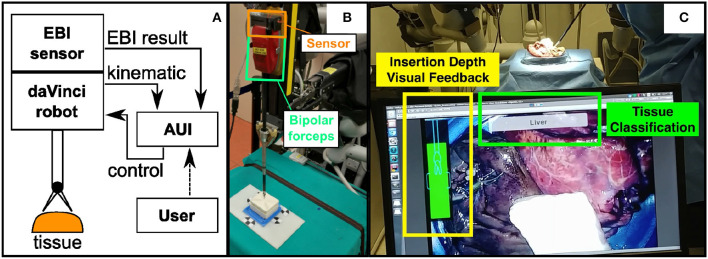
**(A)** The schematic of the proposed system; **(B)** The designed EBI sensor is mounted on the top surface of a standard Maryland bipolar forceps; **(C)** The Advanced User Interface is designed to control the EBI sensing and display the identification results.

The proposed system can be seen in Figure 1A. The EBI sensing technology and the AUI are designed and integrated into a da Vinci Research Kit (dVRK) for providing tissue identification (Kazanzides et al., 2014). The EBI sensor, which is integrated to the bipolar robotic forceps, is mounted on the Patient Side Manipulator (PSM) of the da Vinci robot. It measures the electrical bio-impedance of the tissue contacting the tool tips. The measured values are sent to the connected computer for signal processing and tissue identification. The AUI runs on the same computer and uses the master console stereo-viewers to provide visual guidance to the user during the EBI sensing procedure. In addition, the tissue identification results and the augmented-reality labels are displayed on the AUI.

Figure 1B shows the hardware of the EBI sensor directly mounted on a bipolar robotic tool from the da Vinci surgical robot. In this study, we choose a standard Maryland bipolar forceps (Ref. 400172) as an example. More details related to the EBI sensing will be described in section 2.2.

As shown in Figure 1C, the AUI is designed to show three different pieces of information in real time. Firstly, it shows the result of the classification in text form. Secondly, a tri-dimensional point on the measurement site is created on the AUI to mark the classified tissue. Thirdly, a visual feedback of the insertion depth which is derived from the robotic kinematic information is displayed for guiding the user during the EBI measurement. A detailed description of the AUI is provided in section 2.3.

### 2.2. The Electrical Bio-impedance Sensing System

#### 2.2.1. EBI Sensing Principle

In this study, the two jaws of the bipolar forceps are exploited as an electrode pair for EBI sensing. To measure the electrical impedance of a tissue sample, an excitation alternate voltage *U* is applied through the tissue sample. By measuring the feedback current *I*, the impedance of the tissue *Z* can be computed as the ratio of voltage to current, and furthermore *I* can be computed as an integration of the current density (*J*_*tot*_):

(1) $$\begin{array}{r} Z=\frac{U}{I}=\frac{U}{\int J_{tot}} \end{array}$$

The total current density (*J*_*tot*_) includes two components: the real part *J*_*c*_ and the virtual part *J*_*d*_, and it can be computed as

(2) $$\begin{array}{r} J_{tot}=J_{c}+J_{d}=\sigma E-i\omega \varepsilon E \end{array}$$

where σ and ε represent the conductivity and the permittivity of the contacted material, *E* is the electric field, and ω is used to denote the excitation frequency. Therefore, the modulus of the measured EBI value |*Z*| can be calculated as

(3) $$\begin{array}{r} |Z|=\frac{\left|U\right|}{\left|\sigma -i\omega \varepsilon |\times |\int E\right|} \end{array}$$

In this study, the modulus |*Z*| is used for the tissue identification, which is a function of the excitation voltage |*U*|, the material electric characteristics (|σ−*iωε*|) and the electric field generated by the bipolar forceps (|∫*E*|).

**Figure 2:**
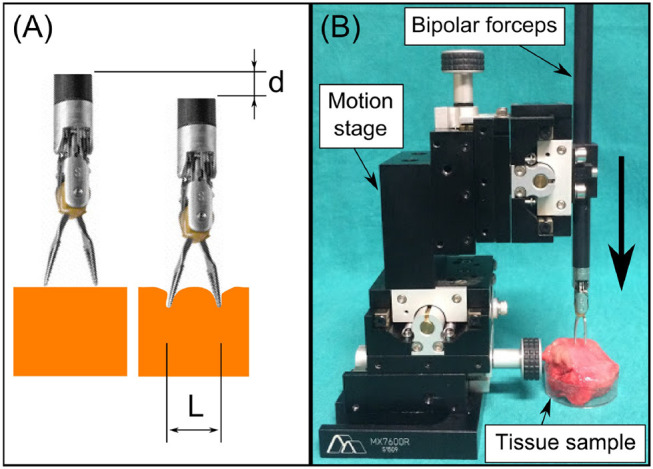
**(A)** We used *L* and *d* to estimate the electrodes configuration and thus the electric field. **(B)** The system setup for measuring EBI of the four tissue types with different *L* and *d*.

In Equation (3), the modulus |σ−*iωε*| represents the electric characteristics of the bio-material when the applied voltage *U* has a constant frequency ω. The electric field *E* depends only on the electrodes' geometry such as electrode sizes and intra-distance, which is explained in the study of Martinsen and Grimnes (2011). Since the jaw opening distance *L* and the pressing depth on the tissue *d* can significantly impact the generated electrical field (see Figure 2A), these two parameters are acquired and controlled in real time during the EBI measurement. We provide the detailed characterization of the EBI sensing system with these two acquisition parameters considered below.

#### 2.2.2. Design the EBI Sensor

As shown in Figure 1B, the EBI sensor's compact dimensions (14 × 20 × 64 mm) enable it to be mounted directly on the top surface of a standard da Vinci surgical tool. The sensor measures the EBI of the tissue contacting its jaws through the proximal end of the forceps, using the same electric connections already used for bipolar electrification.

The EBI sensor electronic design is based on an electrical impedance converter (AD5933, Analog Devices, Norwood, USA) and a micro-controller (Atmega328P, Atmel Inc., USA). The EBI sensor is connected to the master computer via USB. Therefore, the user can control the master computer to set the excitation frequency of the EBI sensor (up to 100 kHz), command the EBI sensor to sleep or request the sensor to stream the measured data |*Z*|.

In this study, the excitation frequency of the EBI sensor was set to 100 kHz for the tissue measurement for two considerations. First, a higher sampling rate could be achieved by executing a higher excitation frequency (Cheng et al., 2017). The system can have a 50 Hz sampling rate with a 100 kHz excitation frequency to ensure the EBI sensing in real time. Second, in their previous studies (Rigaud et al., 1995; Kalvøy et al., 2009) illustrated that the EBI modulus of most tissue types are easier to be distinguished when a higher frequency is applied. This is because at higher excitation frequencies (f > 50 kHz), the cell membrane are electrically shortened and the measured impedance can give information of both intracellular impedance and extracellular impedance, leading to a better description of the tissue's electric properties according to the study of Kyle et al. (2004). Therefore, 100 kHz is chosen to be the excitation frequency for the EBI sensing.

To characterize the measurement performance of the proposed sensor, the EBI system was calibrated using several resistors of known values ranging from 786 Ω to 8.2 kΩ, which covers the expected range for biological tissue electrical impedance. The error rate was found to be 0.59% in average, and the maximum error was found to be 1.2%. For a more detailed analysis of the measurement performance of the proposed system please refer to Cheng et al. (2016, 2018).

#### 2.2.3. Tissue Classification Protocol

A classifier was designed to identify the tissue in contact with the forceps's tip. For this, we first collected data sets of the |*Z*| values of different tissue types with different *d* and *L* (Figure 2A). Then a statistical model was used to fit the collected data set for each tissue type. When the system is used for tissue identification, Mahalanobis distances between the new acquired value and the distributions of different tissue types are calculated. By seeking the shortest Mahalanobis distance, the tissue type can be estimated.

The setup for collecting data from different tissue types is shown in Figure 2B. The EBI sensor was connected and mounted to the bipolar robotic forceps, which was then fixed on the 4th stage of the motion stage (Siskyou, USA). The jaw opening distance of the forceps *L* was adjusted from 2 to 8 mm with 2 mm increments. The pressing depth *d* was controlled by tuning the 4th stage manually. The EBI measurements were collected with *d* from 2 to 4 mm in every 1 mm, because the results of *d* = 0 and 1 mm showed large standard deviation due to unstable contact. For each measurement, the collected data *x* includes the modulus of EBI |*Z*| and the corresponding *L*, *d* values:

(4) $$\begin{array}{c} x={\left[L,d,|Z|\right]}^{T} \end{array}$$

Subsequently, for each tissue type Θ_*i*_, a multivariate normal distribution was used to fit the collected data: Θ_*i*_ ~ *N*(μ_*i*_, Σ_*i*_), where μ is the mean value and Σ is the covariance matrix.

During the application, the sensing system and the forceps are integrated to the da Vinci Research Kit (dVRK) described in Kazanzides et al. (2014). The user controls the forceps to press on the tissue manually via the surgeon console. The EBI sensor measures the impedance of the touching material at the tool tip continuously. Initially, the forceps is exposed in the air and the measured |*Z*| is close to infinity. When the tool tip starts touching a tissue, a relatively small |*Z*| is detected and *d* is set to 0 at this moment. Assuming the tool forceps pressing direction is vertical, the *d* value can be derived from the dVRK kinematic information. Under the assumption that the tool is touching the tissue along the normal direction with respect to the surface, the computed *d* value is a reasonable approximation of an actual touching depth *d*.

As mentioned above, we only use the data with *d* from 2 to 4 mm. When the *d* value is within this range, the EBI value |*Z*|, the depth *d* and the jaw opening distance *L* (acquired from the dVRK directly) are sent to the master computer for tissue type identification. In order to reduce the noise level, the newly collected value $\tilde{x}$ is processed with a low pass filter by averaging every 5 continuous values. Then the Mahalanobis distances between $\tilde{x}$ and the distributions of different tissue types Θ_*i*_ are calculated as

(5) $$\begin{array}{r} D_{i}\left(\tilde{x}\right)=\sqrt{{\left(x-\mu_{i}\right)}^{T}\Sigma_{i}^{-1}\left(x-\mu_{i}\right)} \end{array}$$

The tissue type under measure is estimated to be Θ_*j*_ if the Mahalanobis distance to it is the shortest.

(6) $$\begin{array}{r} \tilde{x}\in \Theta_{j},\text{if}\;j=\arg\underset{i}{\min}\left\{D_{i}\left(\tilde{x}\right)|i=1,2,\ldots ,c\right\} \end{array}$$

### 2.3. The Advanced User Interface

**Figure 3:**
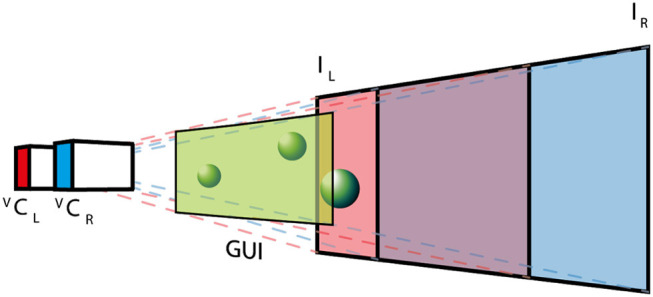
The AUI virtual environment: two virtual cameras (^*V*^*C*_*L*_ and ^*V*^*C*_*R*_) record a textured plane each, where the textures (*I*_*L*_ and *I*_*R*_) are the images obtained with the real stereo-endoscope. The GUI is placed close to the cameras, while 3D objects are positioned between the textured planes and the GUI.

The AUI is based on the Unity3D (Unity Technologies, USA) cross platform virtual reality framework, which provides a straightforward way to create complex computer graphics applications. This framework is based on C# programming language and provides an extensible architecture based on external plug-ins. The integration with dVRK is based on the Robotic Operating System (ROS) interface by creating a C++ native plugin, which exposed required ROS commands to C# Unity scripts. The virtual environment (see Figure 3) replicates the real surgical setup. In the virtual world, the real stereo-endoscope is modeled by two juxtaposed cameras (Virtual Camera Left, ^*V*^ C_L_, and Virtual Camera Right, ^*V*^ C_R_) that record a textured plane each. The two textures (Image Left, *I*_*L*_, and Image Right, *I*_*R*_) are mutually visible by the virtual cameras and they are continuously updated with the images recorded by the dVRK stereo-endoscope during the experimental procedure. The virtual environment preserves the depth perception of the surgical scene and allows the projection of additional information (e.g., user interfaces, 3D models or point-clouds). The graphical user interface (GUI) is placed close to the virtual cameras, such that it can be seen in both fields of views without being occluded by other objects as shown in Figure 3.

**Figure 4:**
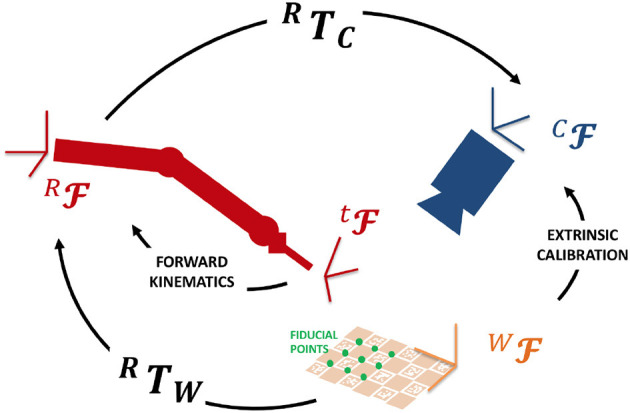
The coordinate transformations for registering the tool tip location in the camera view.

In addition, the projective transformation between the surgical tool tip and the camera reference frames are computed so that punctual EBI information can be overlapped to the endoscopic images in the AUI. To achieve this goal, a registration procedure based on the hand-to-eye calibration method proposed by Strobl and Hirzinger (2006) was implemented. Figure 4 shows the involved reference *Frames* (F) and *Transformations* (T). We selected a number (*N* = 15) of fiducial points on a ChArUco board [i.e., a checkerboard augmented with fiducial visual markers introduced by Romero-Ramirez et al. (2018)]. These points have known coordinates in the *World Frame* (^*W*^F) (i.e., the ChArUco board Frame). Then, we set the board in the endoscopic camera field of view, so that the *Camera Frame* (^*C*^F) can be registered to the *World Frame* by online extrinsic calibration of the camera (finding ^*C*^T*_W_*). We placed the tip of the robotic tool on each fiducial point of the ChArUco board and we recorded the corresponding coordinates in the *Robot Base Frame* (^*R*^F) by computing the Forward Kinematics. Quaternion matching method was applied to estimate the transformation between the *Robot Base Frame* and the *World Frame*: ^*R*^T*_W_*. Finally, the transformation between the *Camera Frame* and the *Robot Base Frame* is derived as ^*C*^T_*R*_ = ^*C*^T_*W*_ · ^*W*^T_*R*_. Thanks to this registration procedure, the position of the tool tip is known in the AUI frame and can be exploited to display the EBI results in the field of view.

The registration is assessed by computing the average positional error of the control points in and out of the calibration board plane (total number of points = 50). The positional error is defined as the Euclidean distance between the points in the world frame and the position calculated by the robot's forward kinematics, and it is found to be 4.1 mm.

As mentioned in section 2.2, the EBI measurement depends on several variables including the jaw opening distance *L* and the insertion depth *d*, which are related to different current densities applied to the tissue and thus result in different EBI measurements. Since *L* is accessible from the robot joint encoders, this parameter is directly exploited for the tissue classification. In addition, the contacting depth *d* is required to be controlled as mentioned in section 2.2.3. Therefore, the correct position of the surgical tool with respect to the tissue surface and its required range are provided on the AUI.

After the tissue identification is done, the identified tissue type is shown in the text box of the AUI. In addition, a dot is marked in a predefined color on the AUI to represent the corresponding tissue type. In this way, we can display the EBI measurements directly on the measurement site, which makes data interpretation intuitive, avoiding increasing the surgeon's cognitive load. In addition, the surgeon is able to disable the AUI data visualization at any time during the operation.

## 3. Experimental Evaluation

### 3.1. Experimental Design

To evaluate the designed system, three types of *ex vivo* porcine tissues including abdominal muscle, liver and lung were selected, which simulated the common environment of an RMIS procedure. The tissue samples were obtained from a butcher shop and most likely belonged to different animals. Surgeons often operate in ambiguous environments containing these three types of tissue, thus one application of the proposed system is to provide real time guidance to enhance the surgeon's awareness of the surgical environment.

In total, 18 pieces of porcine tissues were prepared (6 pieces for each tissue type), among which 9 pieces were used for training the statistical models as described in section 2.2.3 and the other 9 pieces were used for system evaluation.

As for evaluating the system, three tests were conducted on three random places of each tissue sample, resulting in 27 tests in total. For each test, the experimenter manipulated the bipolar forceps with integrated EBI sensor to touch the tissue samples through the console of the da Vinci robot. During the pressing of the tissue, the experimenter was guided to reach the required depth (2–4 mm) by the AUI as *d* was updating in real time. The value of the forceps jaws opening distance *L* was also computed in real time based on the jaw opening angle provided by the robotic system. The evaluation of the designed system considers the identification result which is shown on the text box of the AUI. A mark in the corresponding color of the tissue type is added to the position of the tool tip: red dots for lung tissues, blue dots for liver tissues and green dots for muscle tissues.

### 3.2. Experimental Results

**Figure 5:**
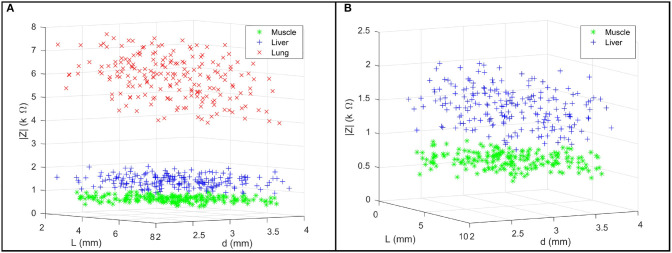
**(A)** For each tissue type, a group of 200 points are used to describe the multivariate normal distribution of the collected EBI measurements |*Z*|, the jaw opening distance *L*, and the pressing depth *d*. **(B)** A magnified view of the data points of muscle and liver.

We firstly present the statistical model of three tissue types Θ_*i*_ as shown in Table 1. In Figure 5A, 200 points were generated to describe the statistical model of each tissue type including the EBI measurement |*Z*| and the *L* and the *d* used for the measurement. Figure 5B provides a magnified view for better showing the normal distributions of muscle and liver. Given 95% confidence (±2σ), the experimental results prove that these three tissue types can be successfully classified by the proposed sensing system since there are no intersections among them.

**Table 1:** For each tissue type, a normal distribution was used to describe the measurements with *d* from 2 to 4 mm.

**Tissue**	**μ**	**Σ**
Muscle	[5, 3, 667.5]^*T*^	$\left[\begin{array}{ccc} 4.81 & 0 & 150.24\\ 0 & 0.67 & -86.84\\ 150.24 & -86.84 & 3.14E4 \end{array}\right]$
Liver	[5, 3, 1464.1]^*T*^	$\left[\begin{array}{ccc} 4.75 & 0 & 360.44\\ 0 & 0.67 & -178.13\\ 360.44 & -178.13 & 1.43E5 \end{array}\right]$
Lung	[5, 3, 5878.3]^*T*^	$\left[\begin{array}{ccc} 5.26 & 0 & 398.25\\ 0 & 0.67 & -529.18\\ 398.25 & -529.18 & 1.20E6 \end{array}\right]$

**Figure 6:**
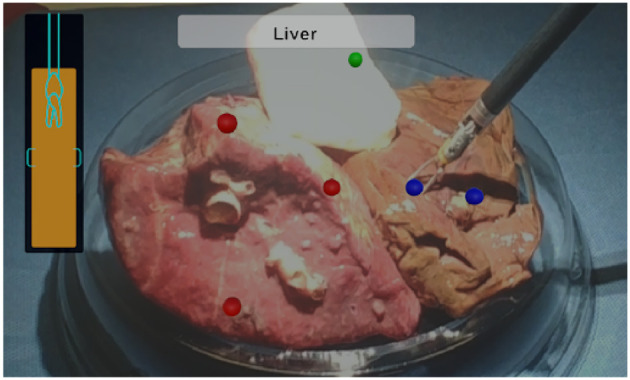
The AUI visualization components: the lateral bar provides a visual feedback of the insertion depth and it turns green when the optimal distance is reached; the upper central box contains the classification outcome in text form; marks in corresponding color are added to the view to indicate the tissue types.

Regarding the test results, the AUI demonstrated to be able to show the *d* value in real time (9.1 Hz), guiding the user to press the forceps on the tissue in the required depth range for the EBI sensing. In addition, the EBI sensor proved to be capable of measuring the EBI of the tissue being touched and to identify the tissue type accurately. Among all 27 tests, the designed system could successfully identify the tissues in all the cases. Finally, the identified tissue types were successfully shown on the AUI text box and corresponding marks were added to the AUI over the sensing positions as shown in Figure 6.

## 4. Discussion

A novel tissue identification method was proposed and designed by sensing the EBI of tissue touched by a bipolar forceps. As demonstrated by the experimental results, by controlling the pressing depth and the jaw opening distance, the EBI sensing system can identify different tissue types such as muscle, lung and liver with 100% accuracy. According to Equation (3), the measured EBI value |*Z*| actually represents the tissue electric property |σ − *iωε*|, given the electric field generated by a specific setting of pressing depth and jaw opening distance. In fact, the electrical property of a specific tissue type reflects the intracellular-extracellular-membrane relationship according to Martinsen and Grimnes (2011). Therefore, different tissue types can be distinguishable based on their EBI value since their cell compositions are different.

In addition, the AUI provides integrated AR data visualization and indications of the surgical tool positioning to obtain consistent measurements. The visual feedback on the tool pressing depth assures a more accurate tissue identification. Also, it allows to map the surgical scene with information of the tissues touched by the tool tip. In terms of the positioning accuracy of the marks, the calibration method allows to obtain an accuracy (reprojection error) in the order of millimeters. The main reason for the limited accuracy obtained is probably due to the intrinsic error of the dVRK forward kinematics (Kwartowitz et al., 2006).

This study still have some limitations. One of them is related to the error in estimating the pressing depth *d*. In this study, the vertical displacement of the forceps was used to estimate the pressing depth without considering the tissue indentation and movement. Fortunately, the parameter *d* in this study was controlled to be relatively small, and thus we expect a minimum impact of this error when more generic acquisition conditions will be used. Another limitation is that interstitial fluids or blood on the organ surface can easily contaminate the acquired EBI value. Therefore, it is necessary to remove liquids from the tissue surface (for instance with suction tool) to obtain meaningful EBI values.

## 5. Conclusion

This study presents the design of novel sensing system that can be easily integrated into a commercial surgical robotic system for identifying different tissue types. The system includes two components: an EBI sensor which is connected to a bipolar forceps for measuring the electric property of the touching tissue; and an AUI for guiding the EBI measurement and displaying the tissue types. The designed system has potential to be very helpful given its remarkable capability of on-site tissue identification in real time, especially when the visual feedback provided by an endoscope fail to allow such identification (e.g., in case of blurred camera or smoke in the field of view). Most importantly, the system could potentially help in tumors resections because Laufer et al. (2010) have demonstrated that EBI sensing is able to discriminate between healthy and cancerous tissues, which is difficult to be achieved based on only visual inspection.

Future work will focus on the following aspects. We plan to extend the system evaluation and characterization by considering more tissue types in different conditions (e.g., with different moisturize levels, considering the presence of blood or other physiological liquid on the surface). In order to identify similar tissue types or to discriminate between healthy and cancerous tissues, multiple frequencies can be applied for improving the EBI sensing. Also, advanced machine learning algorithms will be applied on the improved EBI sensing for obtaining robust tissue classification. In addition, 3D reconstruction based on stereo endoscope images will be integrated into the current system for a more accurate pressing depth estimation, especially when the surgical instrument is contacting the tissue with a generic orientation during EBI measurements. As for the AUI, the implementation of tracking algorithms can be improved in order to adjust the position of the classification dots in case of tissue displacement.

## Data Availability

The raw data supporting the conclusions of this manuscript will be made available by the authors, without undue reservation, to any qualified researcher.

## Author Contributions

DD and ZC provided the concept. ZC developed the EBI sensing system. DD, TC, and SF setted up the dVRK. SF and AM developed the AUI. DD, SF, and AM performed the experimental evaluation with da Vinci Robot. ZC, DD, SF, and AM contributed to the manuscript drafting. DC, GF, ED, LM, and PF supervised the research development and revised the manuscript.

### Conflict of Interest Statement

The authors declare that the research was conducted in the absence of any commercial or financial relationships that could be construed as a potential conflict of interest.

## References

[B1] Cheng Z. Davies B. Caldwell D. Mattos L. (2018). A new venous entry detection method based on electrical bio-impedance sensing. Ann. Biomed. Eng. 46, 1558–1567. 10.1007/s10439-018-2025-7 29675812

[B2] Cheng Z. Davies B. L. Caldwell D. G. Barresi G. Xu Q. Mattos L. S. (2017). A hand-held robotic device for peripheral intravenous catheterization. Proc. Inst. Mech. Eng. H. 231, 1165–1177. 10.1177/0954411917737328 29059005

[B3] Cheng Z. Davies B. L. Caldwell D. G. Mattos L. S. (2016). A venipuncture detection system for robot-assisted intravenous catheterization, in 2016 6th IEEE International Conference on Biomedical Robotics and Biomechatronics (BioRob) (Singapore: IEEE), 80–86.

[B4] Elhage O. Murphy D. Challacombe B. Shortland A. Dasgupta P. (2007). Ergonomics in minimally invasive surgery. Int. J. Clin. Pract. 61, 186–188. 10.1111/j.1742-1241.2006.01243.x 17263704

[B5] Guru K. A. Shafiei S. B. Khan A. Hussein A. A. Sharif M. Esfahani E. T. (2015). Understanding cognitive performance during robot-assisted surgery. Urology 86, 751–757. 10.1016/j.urology.2015.07.028 26255037

[B6] Kalvøy H. Frich L. Grimnes S. Martinsen Ø. G. Hol P. K. Stubhaug A. (2009). Impedance-based tissue discrimination for needle guidance. Physiol. Meas. 30, 129–140. 10.1088/0967-3334/30/2/002 19136732

[B7] Kalvøy H. Sauter A. R. (2016). Detection of intraneural needle-placement with multiple frequency bioimpedance monitoring: a novel method. J. Clin. Monit. Comput. 30, 185–192. 10.1007/s10877-015-9698-25902898 PMC4792358

[B8] Katić D. Schuck J. Wekerle A.-L. Kenngott H. Müller-Stich B. P. Dillmann R.. (2016). Bridging the gap between formal and experience-based knowledge for context-aware laparoscopy. Int. J. Comput. Assist. Radiol. Surg. 11, 881–888. 10.1007/s11548-016-1379-2 27025604

[B9] Kazanzides P. Chen Z. Deguet A. Fischer G. S. Taylor R. H. DiMaio S. P. (2014). An open-source research kit for the da vinci surgical system, in IEEE International Conference on Robotics and Automation (ICRA) (Hong Kong), 6434–6439.

[B10] Konstantinova J. Jiang A. Althoefer K. Dasgupta P. Nanayakkara T. (2014). Implementation of tactile sensing for palpation in robot-assisted minimally invasive surgery: a review. IEEE Sens. J. 14, 2490–2501. 10.1109/JSEN.2014.2325794

[B11] Kwartowitz D. M. Herrell S. D. Galloway R. L. (2006). Toward image-guided robotic surgery: determining intrinsic accuracy of the da Vinci robot. Int. J. Comput. Assist. Radiol. Surg. 1, 157–165. 10.1007/s11548-006-0047-3 20033594

[B12] Kyle U. G. Bosaeus I. De Lorenzo A. D. Deurenberg P. Elia M. Gómez J. M.. (2004). Bioelectrical impedance analysis–part I: review of principles and methods. Clin. Nutr. 23, 1226–1243. 10.1016/j.clnu.2004.06.004 15380917

[B13] Laufer S. Ivorra A. Reuter V. E. Rubinsky B. Solomon S. B. (2010). Electrical impedance characterization of normal and cancerous human hepatic tissue. Physiol. Meas. 31, 995–1009. 10.1088/0967-3334/31/7/009 20577035

[B14] Martinsen O. G. Grimnes S. (2011). Bioimpedance and Bioelectricity Basics. Academic Press.

[B15] Meershoek P. KleinJan G. H. van Oosterom M. N. Wit E. M. van Willigen D. M. Bauwens K. P.. (2018). Multispectral-fluorescence imaging as a tool to separate healthy from disease-related lymphatic anatomy during robot-assisted laparoscopy. J. Nucl. Med. 59, 1757–1760. 10.2967/jnumed.118.211888 29777008

[B16] Moccia S. Wirkert S. J. Kenngott H. Vemuri A. S. Apitz M. Mayer B.. (2018). Uncertainty-aware organ classification for surgical data science applications in laparoscopy. IEEE Trans. Biomed. Eng. 65, 2649–2659. 10.1109/TBME.2018.2813015 29993443

[B17] Pavan N. Zargar H. Sanchez-Salas R. Castillo O. Celia A. Gallo G.. (2016). Robot-assisted versus standard laparoscopy for simple prostatectomy: multicenter comparative outcomes. Urology 91, 104–110. 10.1016/j.urology.2016.02.032 26948530

[B18] Penza V. Ortiz J. De Momi E. Forgione A. Mattos L. (2014). Virtual assistive system for robotic single incision laparoscopic surgery, in 4th Joint Workshop on New Technologies for Computer/Robot Assisted Surgery (Genova), 52–55.

[B19] Rigaud B. Hamzaoui L. Frikha M. Chauveau N. Morucci J.-P. (1995). *In vitro* tissue characterization and modelling using electrical impedance measurements in the 100 Hz-10 MHz frequency range. Physiol. Meas. 16, A15–A28. 10.1088/0967-3334/16/3A/002 8528113

[B20] Romero-Ramirez F. J. Muñoz-Salinas R. Medina-Carnicer R. (2018). Speeded up detection of squared fiducial markers. Image Vis. Comput. 76, 38–47. 10.1016/j.imavis.2018.05.004

[B21] Schoevaerdts L. Esteveny L. Gijbels A. Smits J. Reynaerts D. Vander Poorten E. (2018). Design and evaluation of a new bioelectrical impedance sensor for micro-surgery: application to retinal vein cannulation. Int. J. Comput. Assist. Radiol. Surg. 14, 311–320. 10.1007/s11548-018-1850-3 30141126

[B22] Simorov A. Otte R. S. Kopietz C. M. Oleynikov D. (2012). Review of surgical robotics user interface: what is the best way to control robotic surgery? Surg. Endosc. 26, 2117–2125. 10.1007/s00464-012-2182-y 22350236

[B23] Strobl K. H. Hirzinger G. (2006). Optimal hand-eye calibration, in 2006 IEEE/RSJ International Conference on Intelligent Robots and Systems (Beijing: IEEE), 4647–4653.

